# Determinants of journal choice among Nigerian medics

**DOI:** 10.11604/pamj.2015.21.148.6534

**Published:** 2015-06-24

**Authors:** Nwhator Solomon Olusegun, Agbaje Maarufah Olayinka, Soroye Modupe, Isiekwe Gerald Ikenna

**Affiliations:** 1 Department of Preventive & Community Dentistry, Faculty of Dentistry, College of Health Sciences Obafemi Awolowo University, Ile-Ife, Nigeria; 2 Department of Child Dental Health, Lagos State University College of Medicine, Lagos, Nigeria; 3 Department of Child Dental Health, Faculty of Dental Sciences, University of Lagos, Nigeria

**Keywords:** Journal choice, medics, academicians, Nigeria

## Abstract

**Introduction:**

Despite the well-known maxim "publish or perish" among academicians, productivity remains low in Nigeria. There are barriers to academic writing which must be identified and addressed. Even after addressing those barriers, authors are faced with another dilemma-where to publish. It was the concern of the authors to evaluate perceived barriers to academic writing and the determinants of journal choice among Nigerian academics. They also attempted to evaluate the determinants of journal choice and perceived barriers to academic writing among Nigerian academicians. Respondents were academicians used in the context of this study to mean anyone involved in academic writing. Such persons must have written and published at least one paper in a peer-reviewed journal in the preceding year to be included in the survey. An online-based self-administered questionnaire.

**Methods:**

An online structured and self-administered questionnaire-based cross sectional survey of Nigerian medical academicians was conducted over a period of one year using a Google-powered questionnaire. The questionnaire assessed the determinants of journal choice, perceived barriers to publications, number of publications in the preceding year as a measure of academic productivity and the highest publication fee authors were willing to pay.

**Results:**

Of the over 500 email request sent, a total of 200 academicians responded (response rate of 40%). The male and female distribution was 120 and 80 respectively. The highest number of respondents were lecturer 1 and senior lecturers (or junior faculty) (69.5%) however the senior faculty had the higher number of publications in the preceding year. Indexing (35.5%) was the most important determinant of journal choice whilst ease of submission (2.1%) was the least. Unfriendly environment (46%) was the most perceived barrier to publication. Though, majority (88.5%) of the respondents were willing to pay up $300 as publication fees, twice as many junior faculty members (28%) were willing to pay more than $300 as publication fee when compared with professors (12.5%). About 140 of the respondents (70%) were doctors/dentists.

**Conclusion:**

In this study, the major determinant of journal choice among Nigerian medics is journal indexing and unfriendly environment appears to be the major perceived barrier to publication. Encouraging a friendly and conducive environment in the universities will impact positively in academic productivity amongst Nigerian faulty members.

## Introduction

Just as with any topic in the realm of academia, the subject of the true measure of academic productivity has remained an enigma. The junior faculty grapples with the realization that he must dance to the same unwanted tune-"publish or perish" that he wishes was non-existent, to achieve his dreams of becoming a celebrated academic. The measure of productivity - the number and quality of publications-remains the focus despite obvious challenges. While the junior faculty brace up to a forced love of the unpleasant tune, the older respected faculty might look back with hindsight agreeing with Brew [1], that the focus on publications as an outcome measure of productivity is excessive. Brew's position might be further strengthened by the fact that government policy is influenced more by affluence than by the thinking of the majority [2]. Furthermore, no sooner the junior faculty decides to concentrate on this "tune", than he meets a rude shock that his conclusions may not be as useful they appear. A text on policy for instance reads "RCTs are by no means the panacea they are sometimes presented as. In medicine they are routinely overturned by new evidence, and they are particularly ill-suited to many fields of social policy" [3]. "Do academics want to publish?" and do academics encounter challenges and barriers in their attempt at publishing in order not to perish? Despite the obvious answers to the two rhetoric questions, inadequate publication output remains the albatross of many academics with figures as low as 0.4 papers per year in South Africa [4]. The Nigerian situation is even worse with the conclusion that she has "regressed in many fields of science" [5]. Nigeria was ranked second to South Africa in a general publication output measured over an 11-year period [6]. In a separate study, Nigeria lagged behind Ghana, Senegal, Niger, Benin and other much smaller countries [7]. Barriers are natural to every field of human endeavour. Barriers to academic writing and publishing range from the fear of rejection, through unfavourable reviews to lack of time and other factors as identified in the well-cited systematic review by McGrail et al., [8]. These barriers have been grossly under-researched among Nigerian academics. The purpose of this study therefore, was to assess the barriers to academic writing among Nigerian academics and to evaluate the determinants of journal choice when Nigerian authors eventually overcome the inertia/barriers on their path to becoming celebrated academics. In conducting this study, the authors attempted to capture the influence of several variables on the productivity of Nigerian academics. Admittedly, productivity was judged (in a very limited sense) as the number of peer-reviewed publications in the preceding 12 months to the study. While, the authors accept this as a limited yet objective test, they chose to adopt it just as schools have continued to depend on examinations for academic assessments despite the age-long dictum that examinations are not a true test of knowledge.

## Methods

**Research design:** this was an online cross-sectional survey of academics in Nigeria using an online Google-powered questionnaire.

**Sampling:** the sampling method adopted was non-random. Emails of academic staff from all available sources were entered into an online survey system. The respondents cut across academicians of various disciplines. It was online-based, hence there were no institutional boundaries. The online questionnaire was designed such that only respondents who had published at least one paper in the preceding 12 months were allowed to complete the survey.

**Research participants**: participants consisted of 200 academicians who gave consent to participate in an online survey over an eleven month period spanning November 2013 through October 2014. The response rate was low and the 200 participants who eventually responded constituted roughly 40% of the total number of emails sent out through Google drive. Academics were regarded as anyone involved in peer-reviewed academic writing irrespective of their institutional affiliation. Biographic data and responses to various variables were obtained through an online questionnaire preceded by an explanation of the purpose and request for consent to participate. The online protocol sought to obtain responses to various questions relating to sex, age-group, academic level, sponsorship, what participants were willing to pay for publications, perceived barriers to publications. Most questions were closed interspersed with a few open-ended questions as shown in Appendix 1.

**Data analysis**: data entry and analysis were performed with the PASW (SPSS) statistical software with univariate analysis of frequencies. Means and standard deviations were excluded since most data though numeric in nature were grouped at the point of data collection and therefore treated as categorical variables. The main dependent (outcome) measures were perceived barriers to publication, number of peer-reviewed publications in the previous 12 months and maximum amounts participants were willing to pay as article processing fees. These influences of explanatory (independent) variables on the main outcome measures were evaluated using chi-square statistics at 95% confidence level which generated respective p-values. P-values of ≤0.05 were therefore regarded as being statistically significant.

## Results

Of the 200 consenting participants (120 male, 80 female), 164 worked in Universities while 36 worked in hospitals. Only 10 academics (5%) always enjoyed sponsorship of their research work and publication, 53 (26.5%) admitted getting sponsored some of the time while most respondents (68.5%) never received sponsorship for their work.

### Barriers to publication

The most important barrier (92, 46%) was an "unfriendly environment" which was an un-qualified closed response whereas open answers captured under "others" included no time, rejection at first instance, slow review, and high publication costs as responded to by 65 (32.5%) of the respondents. Academic levels had no impact on perceived barriers to publication (p = 0.313). There were strong sex differences (p = 0.001) in perceived barriers to publications. More than twice as many males as females cited lack of interest while about twice as many females than males felt hindered by "other factors" which consisted of time constraints, lack of mentoring, funds and delayed reviews (Table 1). Surprisingly, sponsored academics suffered more from "lack of interest" with almost twice their number (31.5% vs 17.5%) citing lack of interest as a barrier than the academics who never received sponsorship. Conversely, more than twice the number (30 vs 13%) of non-sponsored academics felt hindered by "other factors" which consisted of time constraints, lack of mentoring, funds and delayed reviews. The differences were statistically significant (p = 0.021) (Table 2).

**Table 1:** Impact of sex on perceived barriers to publication

Sex	Male		Female	
Important Factors/considerations	n	%	n	%
Unfriendly environment	57	54.8	36	51.4
Not interested	30	28.8	8	11.4
Others	17	16.3	26	37.1
Total^x^	104	100	70	100

X2= 13.22, df = 2, p-value = 0.001 26 respondents indicated no barriers

**Table 2:** Impact of sponsorship on perceived barriers to publication

	Sponsorship-never		Sponsorship-yes	
Important Factors/considerations	n	%	n	%
Unfriendly environment	63	52.5	30	55.5
Not interested	21	17.5	17	31.5
Others	36	30.0	7	13.0
Total^x^	120	100	54	100

X2= 7.77, df = 2, p-value= 0.021 26 respondents indicated no barriers

### Determinants of journal choice

**Figure 1:**
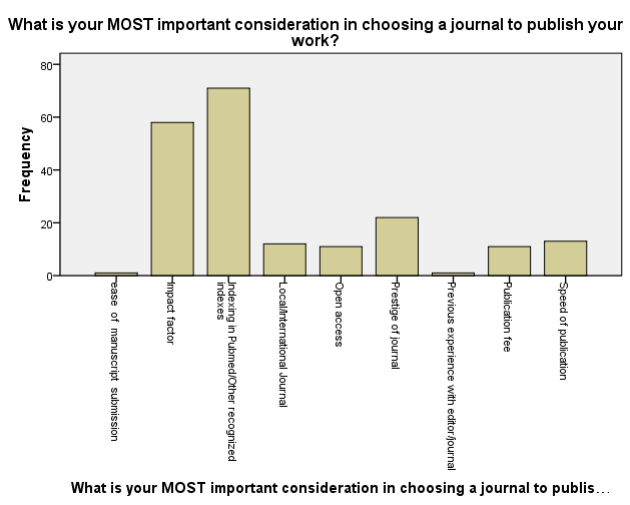
Bar chart of the various which influenced authors’ choice of journal

Overall, indexing (71, 35.5%) was the most important determinant of journal choice followed by impact factor. Previous experience with the editor and the ease of submission were of least importance (2, 1%) (Figure 1. The influence of the sex (p = 0.09) and sponsorship (p = 0.55) of respondents on the choice of where to publish failed to attain statistical significance. While respondents up to lecturer 1 were more concerned about the impact factor and prestige of journals, senior lecturers and professors were concerned about indexing and impact factor. The differences just achieved statistical significance (0.049) (Table 3) but the significance disappears when the lecturers were considered along junior (up to senior lecturer) and senior faculty (professorial) lines X^2^= 1.565, df = 4, p-value = 0.815.

**Table 3:** Impact of academic level on journal choice determinants

Academic level/cadre	Up to lecturer1		Senior lecturer		Professors	
Important Factors/considerations	n	%	n	%	n	%
Impact factor	27	30.7	14	27.5	10	25.0
Indexing	20	22.7	25	49.1	17	42.5
Prestige/Int'l	21	23.9	3	5.9	6	15.0
Pub matters (Speed & Fee)	12	13.6	7	13.8	4	10.0
Open Access + others	8	9.1	2	3.9	3	7.5
Total^x^	88	100	51		40	100

X^2^= 15.54, df= 8, p-value= 0.049

x Only 179 worked in a university system as academy staff

### Academic productivity measured by the number of peer-reviewed publications in the preceding 12months

Only 15 respondents (7.5%) had at least 10 publications to their credit in the preceding 12 months or at least 0.83 publications per month while a vast majority barely managed 1-3 publications in the preceding 12 months or 0.083-0.250 publications per month. Productivity levels were independent of sex (p = 0.439) and receipt of sponsorship (0.1081). Academic productivity was however much higher among the senior faculty because a higher proportion of professors had published at least 7 papers in the preceding 12 months compared with senior lecturers (27.5% vs 17.6%) or (1.5:1). The proportion of professors who had published at least 7 papers in the preceding 12 months was four times higher than the proportion of lecturers 1 and below (27.5% vs 6.8%) or 4:1 (p = 0.0004) (Table 4). The statistical significance remained (howbeit less strongly) even after considering the authors along the lines of junior and senior faculty (p = 0.02).

**Table 4:** Impact of academic level on number of publications in preceding 1 year

Academic level/cadre	Up to lecturer 1		Senior lecturer		Professors	
No of publications in the preceding 12 months	n	%	n	%	n	%
1-3 publications	65	73.9	24	47.1	15	37.5
4-6 publications	17	19.3	18	35.3	14	35.0
≥ 7publications	6	6.8	9	17.6	11	27.5
Total^x^	88	100	51	100	40	100

X^2^= 20.44, df= 4, p-value= 0.0004

x Only 179 worked in a university system as academy staff

^xx^ All tables satisfy Cochran's criteria with none of the cells having expected values < 5, and with no cells having expected values < 1

### Highest publication fee authors were willing to pay

Most (177, 88.5%) of the authors in this study were willing to pay up to $300 as publication fees irrespective of sex (p = 0.09) and receipt of sponsorship (p= 0.90). Twice as many junior faculty members were willing to pay more than $300 publication compared with professors (28,25.7% vs 5,12.5%). The difference however failed to achieve statistical significance (p = 0.1268 (Fisher's Exact)). All Chi-square calculations satisfied Cochran's criteria that none of the cells have expected values < 5, No cells have expected values < 1. Only significant tables are shown due to space constraints.

## Discussion

Barriers are natural to any human endeavour. Academicians working in a resource-starved environment like Nigeria are not spared from barriers neither are they immune to them. Since this was a simple study with closed mostly "yes", "no" patterned answers, it was not possible to evaluate secondary relationships between different barriers and considerations. For instance, while the study attempted to understand barriers, it only sought to evaluate the "greatest" barrier only rather than evaluate the detailed relationships between the various barriers-for instance evaluating if an author would be more concerned for open access or for impact factor if cost wasn't an issue. It is indeed possible that open access would be the next "big thing" for an author if he had the required funding. For other authors, journal prestige might take the front stage if indexing is taken care of. The authors also gave no option of zero cost to authors who only publish in free journals! Unfortunately, the current study was not powered to make such detailed, multi-level questioning. Admittedly, this design was a bit unfair to the respondents but it provides some baseline data upon which future studies can be built. Now, sponsorship is one big issue in Nigeria and has been recognized as a barrier to productivity and research output [9]. While sponsorship from commercial sponsors is a recognized source of temptation [10, 11], over a decade ago, Okebukola of the Nigerian Universities Commission identified "difficulty in accessing research funds" as one of the major reasons for the declining research productivity in Nigerian tertiary institutions [12]. The question is "how much has changed in the positive direction since his observation"? Beyond sponsorship, an "unfriendly environment" was the weeping child in this study being the cited barrier by 92,46% of authors. Unfortunately, the term "unfriendly environment" was the coinage of the authors and is subject to lots of interpretations. It would be interesting to replicate this study on a greater scale where authors are asked to express what in their concept constitutes an "unfriendly environment." Be that as it may, the sharp sex differences reported here seem expected with twice as many females citing time constraints and related factors than male academics. This might be a reflection of the herculean task of balancing the home front with academia. Our findings corroborate previous studies [13]. However, Omoniyi and Ogunsanmi found no link between sex and stress levels among University staff in Southwest Nigeria [14]. Female lecturers were more satisfied with their jobs in Malaysia [15] while male lecturers were more likely to be considered "research-active" than their female counterparts in British Universities [16]. One paradoxical finding from this study is the reported "lack of interest" barrier which was statistically higher among male authors. The finding corroborates a recent report that Black African men were less interested in research [17] but is at variance to another report that women were less interested in academic careers than men [18].

In a recent report, Wang and Shapira [19] showed that sponsorship is linked to research impact and citations. Yusuf [20] rightly observed that "Constraints hampering the realisation of research goals in the higher education sector include inadequate and irregular funding, poor motivation, poor or obsolete research infrastructure". It is therefore surprising that while those who never received sponsorship complained about lack of time, the sponsored authors cited lack of interest as a barrier to productivity. As important as it appears therefore, sponsorship alone cannot explain the declining productivity of Nigerian academics. The foregoing makes it imperative to further explore the "unfriendly environment" as it might transcend the realms of research sponsorship into those of psychologic and social distractions beyond the controls of the ivory tower. This position appears even more plausible with another shocking finding that sponsorship did not significantly increase academic productivity among this group of Nigerian academicians. As reported by a Saudi Arabian study [21] and as widely believed among the younger faculty in Nigeria, Professors are likely to publish less as they become saddled with administrative responsibilities (anecdotal). Significantly more Professors in the current study were however more likely to be more productive with at least seven papers to their credit in the preceding 12 months. This finding though at variance with the Saudi Arabian study, corroborates a recent Indian study [22]. Nigerian academics are yet to get on-board the open access train. This study shows that authors were much more concerned about indexing and impact factor. Interestingly, Nigerian academics are oblivious of the rising evidence that open access tends to increase the impact factor of journals [23, 24]. The fact that close to 90% of respondents were willing to pay not more than $300 as publication fees makes a clear statement-that cost is an issue though not clearly stated as such in the current research. Whether or not cost had an overriding effect on the other determinants of journal choice remains unclear from this study. **Limitation**: about 140 of the respondents (70%) were doctors/dentists while about 30% were non-medics. The findings should be interpreted with this in mind.

## Conclusion

The major determinants of journal choice among Nigerian academicians appear to be indexing and impact factor and most Nigerian academics are unwilling to pay more than $300 as article publication fees. More importantly, the fact that only 5% of academics got sponsored all of the time is embarrassing for a country that prides herself as the giant of Africa. The authors would therefore suggest that the Nigerian government should be more pragmatic in restoring the glory of the ivory towers through the creation of a "friendly" and conducive environment that would naturally encourage greater research productivity. Within the limits of the strength of the small sample studied here, it looks like "dumping" money in the universities for research without addressing deep-seated barriers will make the dream of greater productivity a mere mirage.
